# Analysis of the Relationship between the Expression Levels of Neutrophil Gelatinase-Associated Lipocalin and Cytokine Genes in Bone Marrow

**DOI:** 10.7150/ijms.62425

**Published:** 2021-07-23

**Authors:** Sungchul Mun, Min-Chul Park, Chi-Hyun Cho

**Affiliations:** 1 Department of Industrial Engineering, Jeonju University, 303 Cheonjam-ro, Wansan-gu, Jeonju-si, 55069, Republic of Korea;; 2 Center for Opto-Electronic materials and devices, Korea Institute of Science and Technology, Seoul, Republic of Korea;; 3 Department of Laboratory Medicine, College of Medicine, Korea University Ansan Hospital, 123, Jeokgeum-ro, Danwon-gu, Ansan-si, Gyeonggi-do 15355, Republic of Korea.

**Keywords:** neutrophil gelatinase associated lipocalin, cytokine, myeloproliferative neoplasm, acute myeloid leukemia, myelodysplastic syndrome, bone marrow

## Abstract

**Background**: Recently, various associations of NGAL with several hematological cancers have been reported. However, given that the regulation of *NGAL* gene expression by cytokines is tissue-specific,* NGAL* expression in relation to those of cytokine genes has not been analyzed in bone marrow (BM) tissue. The purpose of this study was to analyze the association between *NGAL* and 48 cytokine gene expression levels in mononuclear cells (MNCs) of BM at the time of diagnosis of hematological malignancy and to explore the expression pattern of *NGAL* and related cytokine genes in patients with hematological malignancies and controls.

**Methods**: BM MNCs were isolated from 48 patients, who were classified as patients presenting myeloproliferative neoplasm, acute myeloid leukemia, myelodysplastic syndrome, and as controls. *NGAL* and cytokine genes were analyzed using NanoString. Data on hematological parameters were collected from medical records. Single and multiple regression analyses were performed to analyze relationships.

**Results**: Normalized counts of 26 cytokine genes were related to *NGAL* normalized counts, while *STAT3* and *TLR4* normalized counts had the highest explanatory power. The following multiple regression model was developed: *NGAL* normalized counts=4316.825 + 9.056 × *STAT3* normalized counts + 844.226 × *IL5* normalized counts + 17.540 × *TLR1* normalized counts - 28.206 × *TLR2* normalized counts - 42.524 × *IRAK4* normalized counts. In the multiple regression analysis, *STAT3* and *TLR4* normalized counts showed multicollinearity. *NGAL*, *STAT3*, *IL5*, and *TLR4* normalized counts showed similar intergroup patterns.

**Conclusions**: *NGAL* normalized counts was predicted by a multiple regression model, while they showed similar intergroup patterns to *STAT3*, *IL5*, and *TLR4* normalized counts.

## Introduction

Neutrophil gelatinase-associated lipocalin (NGAL), a member of the lipocalin superfamily, is a glycoprotein originally isolated from the secondary granules of human neutrophils 1. While NGAL is useful as a biomarker especially in kidney injury, it is also expressed in other tissues in response to various pathological conditions, such as ischemia, tissue injury, and cancer 1-3. Being a pleiotropic mediator of various inflammatory processes 4, 5, NGAL acted at different levels as a regulator of multiple responses 6. Recently, association of NGAL with several hematologic cancers have been reported 1, 5-8.

A few studies measuring NGAL protein levels in the bone marrow (BM) of patients with hematological cancer reported that NGAL levels were significantly lower in patients with acute myeloid leukemia (AML) and myelodysplastic syndromes (MDS) than in controls 8-10. Given that neutrophilic precursors are the major source of NGAL synthesis and these exist primarily in the BM but not the peripheral blood, such studies have enabled an accurate comparative analysis of NGAL levels according to the hematological malignant disease entity 8.

Nevertheless,* NGAL* expression has not been analyzed in BM aspirate cells of patients with hematological malignancies. In particular, although *NGAL* expression is regulated by various types of inflammatory cytokines, it should be noted that the regulation of *NGAL* expression is tissue-specific 1. Accordingly, analysis of the relationship between NGAL and inflammatory cytokines in BM cells is important for elucidating the mechanism of the regulation of NGAL in the BM.

The purpose of this study was to identify the expression profile of *NGAL* and 48 cytokine genes in mononuclear cells (MNCs) of BM aspirate at the time of the diagnosis of a hematological malignancy using the nCounter system 11 and analyze the relationship between *NGAL* expression and those of 48 cytokine genes. Additionally, we explored the expression patterns of *NGAL* and related cytokine genes in patients with hematological malignancies and controls.

## Materials and Methods

### Sample collection and preparation

The study was approved by the Institutional Review Board of Korea University Ansan Hospital and performed in accordance with the Declaration of Helsinki. Patients were enrolled between May 2018 and July 2019. Informed consent was obtained from all participants (n=48). Aliquots of leftover BM aspirate samples were collected from patients who underwent BM examination for the diagnosis of hematological malignancies. Patients were classified into myeloproliferative neoplasm (MPN), AML, and MDS groups based on the WHO diagnostic criteria, after the BM smear and pathological review. The control group (n=9) consisted of patients with lymphoma without BM involvement (n=7) or normocellular marrow without hematological malignancy (n=2) 8, 9. None of the patients manifested symptoms of active infections, inflammatory diseases, or kidney failure 7.

BM aspirates were collected in BD Vacutainer tubes (Becton Dickinson, Franklin, NJ, USA) containing EDTA and centrifuged (2399 × *g*, 10 min). After centrifugation, BM infranatants (including BM hematopoietic cells) were used for MNC isolation.

### MNC isolation

Isolation of MNCs from BM aspirates was performed using Lymphoprep in SepMate tubes (STEMCELL Technologies, Vancouver, Canada) 12. First, 4.5 mL of Lymphoprep (density gradient medium; density: 1.077 g/mL; STEMCELL Technologies) was added to 15 mL of SepMate tubes and pipetted through the central hole of the insert. The BM aspirate sample was diluted with an equal volume of phosphate-buffered saline (PBS) and mixed gently, then pipetted down the side of the (vertically held) tube. The tubes were then centrifuged (1200 × *g*, 10 min) and the top layer containing the enriched MNCs was poured into a new tube (15-mL conical polypropylene tube; Becton Dickinson), and PBS was added, resulting in a total volume of 14.5 mL. After centrifugation (300 × *g*, 8 min), the supernatant was carefully removed, and 2 mL of PBS was added to the infranatant (including BM MNCs). The solutions were mixed well, and the mixture was transferred to 1.5-mL Eppendorf tubes and centrifuged (400 × *g*, 5 min). The supernatant was then removed and the infranatant and isolated BM MNCs were stored at -80 °C until mRNA extraction.

### mRNA extraction from BM MNCs

Frozen cell pellets were thawed slowly in a pre-chilled bead bath. Then, 1 mL Trizol (#79306-200mL; Qiagen, Valencia, CA, USA) was added to the thawed cell pellets. The mixture was briefly vortexed and incubated for 5 min at 20-24 °C, and 200 μL chloroform (#34854-1L; Sigma Aldrich, St. Louis, MO, USA) was added. The mixture was shaken for 15 s and incubated for 5 min at 20-24 °C. After centrifugation (13,000 × *g*, 15 min, 4 °C), the aqueous phase was transferred to new 1.5-mL tubes and an equal volume of isopropanol was added. The tubes were inverted manually four times, incubated at 20-24 °C for 10 min, and centrifuged (13,000 × *g*, 10 min, 4 °C). The supernatant was discarded. The pellets were washed twice with 1 mL of 70% ethanol and centrifuged (7,500 × *g,* 5 min, 4 °C). RNA pellets were air-dried. RNA was resuspended in 10-20 μL RNase-free water, transferred to new 1.5-mL tubes, and RNA quality was determined using a 2100 Bioanalyzer capillary electrophoresis system (Agilent Technologies, Santa Clara, CA).

### mRNA expression analysis

Extracted RNA samples were run on an nCounter Analysis System (NanoString Technologies Inc., Seattle, WA, USA) according to the manufacturer's instructions. A cytokine panel was used, which included the following genes: *BAX, BCL2L1, CASP8, CRP, CXCL1, CXCL10, CXCL2, CASPASE-1, ETS like transcription factor-1 (ELK1), FOS, IL10, IL11, IL18, IL1A, IL1B, IL1R1, IL1R2, IL2, IL28A/B, IL29, IL3, IL4, IL5, IL6, IL7, IL8, IL9, INFB, IRAK1, IRAK2, IRAK4, interferon regulatory factor 7 (IRF7), MyD88, NF-κB, NFKB1A, NGAL, NLRP3, RAGE, STAT1, STAT2, STAT3, STAT4, TLR1, TLR2, TLR4, TLR5, TNF, TRAF3,* and* TRAF6*.

A reaction mixture containing RNA (5 μL; 100-300 ng), Master Mix (8 μL; reporter CodeSet and hybridization buffer), and capture probeSet (2 μL) was prepared, and the solution was mixed and spun down. It was incubated at 65 °C in a thermocycler (Bio-Rad Laboratories Inc., Hercules, CA, USA) for 16 h (maximum hybridization, 48 h). Samples were then transferred to the preparation station (NanoString Technologies, Inc.) with a prepared nCounter Master Kit and cartridge. After binding the sample to the cartridge, 12 lanes per run of the nCounter prep station were run for approximately 2.5‒3 h. Cartridges were transferred to the Digital Analyzer (NanoString Technologies Inc.) for analysis and scanned on a digital analyzer at 555 fields of view.

As quality checks for raw data, we confirmed the following: (i) Imaging quality control (QC): Field of View>75%; (ii) Binding Density QC: 0.1<Binding Density<2.25; (iii) Positive Control Linearity QC: R2>0.95; (iv) Limit of Detection QC: 0.5 fM positive control probe>2 standard deviation + mean of the negative controls. Data were normalized using the control probes from Codeset. Normalized expression values of *NGAL* and 48 cytokine genes were used for the statistical analysis.

### Clinical data collection

Baseline demographic data and data about hematological parameters, including hemoglobin levels, white blood cell (WBC) counts, neutrophil and platelet counts, and C-reactive protein (CRP) levels, were collected from medical records. The estimated glomerular filtration rate (eGFR) was calculated using the Chronic Kidney Disease Epidemiology Collaboration equation.

### Statistical analyses

Quantitative data are presented as median [quartile1 (Q1), Q3] values. To ascertain that the data were normally distributed, the Shapiro-Wilk test was used. For the statistical analysis of patient demographic features and laboratory parameters, either a parametric or non-parametric test was performed to obtain reliable statistical results. In general, statistical power is higher in parametric tests. However, when collected samples deviated from a normal distribution, non-parametric tests lead to stronger relative power than their parametric counterparts. Consequently, better protection against type II errors relative to parametric tests was ensured. The Kruskal-Wallis H test, a non-parametric counterpart of one-way ANOVA, was conducted on the sample data in which normality was untenable.

For pairwise comparisons when a significant difference was found among groups, Scheffé's post-hoc test or Dunn's pairwise test was applied. In the parametric post-hoc analysis, we applied Scheffé's method because the test is robust for different sample sizes between groups.

A simple regression analysis was performed to analyze the relationship between *NGAL* expression levels and other cytokine gene levels. Multiple regression analysis was performed to simultaneously analyze the relationship between *NGAL* gene counts and all statistically significant cytokine gene normalized counts.

To control the inflated Type I error caused by performing multiple comparisons and multiple simple regressions, a Benjamini-Hochberg false discovery rate (FDR) correction was applied 13-18. The correction method adjusts the* p* values in a simpler but more efficient way than the traditional Bonferroni correction. The adjusted alphas were determined to be 0.018 for pairwise comparisons and 0.026 for multiple simple regressions. The procedure to determine the adjusted alpha has been detailed 13.

In the multiple regression analysis, multicollinearity was also analyzed to identify closely related independent variables. When independent variables presented variance influence factor (VIF) values>10, the independent variables were considered to have multicollinearity. The predictive accuracy of the multiple regression models was assessed using Akaike's information criterion (AIC) and adjusted *R*^2^ values. Statistical significance was set at *p<*0.05. Statistical analyses were performed using SPSS version v25.0 (IBM SPSS Statistics, Armonk, NY, USA).

## Results

### Patient characteristics

BM aspirates were collected from 48 patients with a median age of 62 years (range 22-89 years). All 48 patients were at the initial diagnosis stage. Patient demographics, hematological parameters, and normalized counts of *NGAL* are presented in Table 1. Six variables exhibited significant differences among the four groups. For numerical data, the significant results of the pairwise comparison tests between the groups are shown in Supplementary Table S1.

### Simple regression analysis of the relationship of *NGAL* gene normalized counts with those of cytokine genes

The normalized counts of *NGAL* [median (Q1, Q3)] in BM MNCs (n=48) were 11978.32 (2106.77, 41460.04). Among 48 cytokine genes, simple regression analysis identified 26 significant predictors of *NGAL* normalized counts (Table 2). Table 2 presents the normalized counts of each of the 48 cytokine genes.

### Multiple regression analysis of the normalized counts of *NGAL* and those of other cytokine genes

Using normalized counts of* NGAL* as the dependent variable and 26 significant predictors (normalized counts of 26 cytokine genes) identified in the simple regression analysis as independent variables, multiple regression analysis was performed. Two multiple regression models were developed (Table 3).

(Model 1) *NGAL* normalized counts=4165.283 + 7.676 × *TLR4* normalized counts + 882.859 × *IL5* normalized counts + 6.792 × *STAT3* normalized counts - 27.477 × *TLR2* normalized counts + 16.348 × *TLR1* normalized counts - 35.902 × *IRAK4* normalized counts

(Model 2) *NGAL* normalized counts=4316.825 + 9.056 × *STAT3* normalized counts + 844.226 × *IL5* normalized counts + 17.540 × *TLR1* normalized counts - 28.206 × *TLR2* normalized counts - 42.524 × *IRAK4* normalized counts

**Figure 1:**
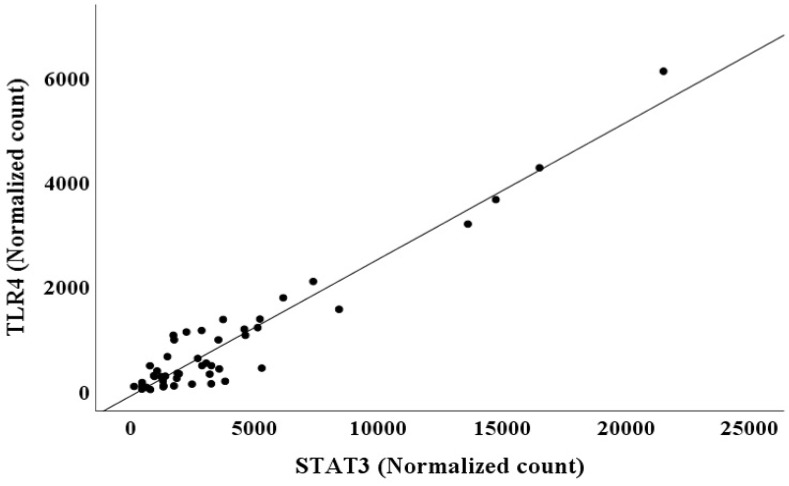
Scatter plot between *TLR4* and *STAT3* normalized counts in bone marrow.

The adjusted *R*^2^ and AIC values are listed in Table 3. The AIC value was lower for Model 1 than for Model 2, and the adjusted *R*^2^ value for Model 1 was higher than that for Model 2. This suggested that model 1 had higher predictive accuracy for *NGAL* normalized counts than model 2 (Table 3) 19, 20. However, multiple regression analysis showed multicollinearity between *TLR4* (VIF=17.277) and *STAT3* (VIF=22.683), two independent variables in model 1 (Table 3). Thus, model 2, in which *TLR4* was removed from the predictor variables, was interpreted as more appropriate for predicting *NGAL* normalized counts than model 1. Because *TLR4* and *STAT3* normalized counts exhibited multicollinearity, a scatter plot between *TLR4* and *STAT3* normalized counts was created (Figure 1).

The relationship between the two variables in BM was further analyzed by simple regression analysis, which showed the following relationship:

*TLR4* normalized counts=-105.521 + 0.262 × *STAT3* normalized counts (R^2^=0.919, *p<*0.001)

### *NGAL*, *STAT3*, *IL5*, and *TLR4* normalized counts in hematological malignant disease groups compared to the control

**Figure 2:**
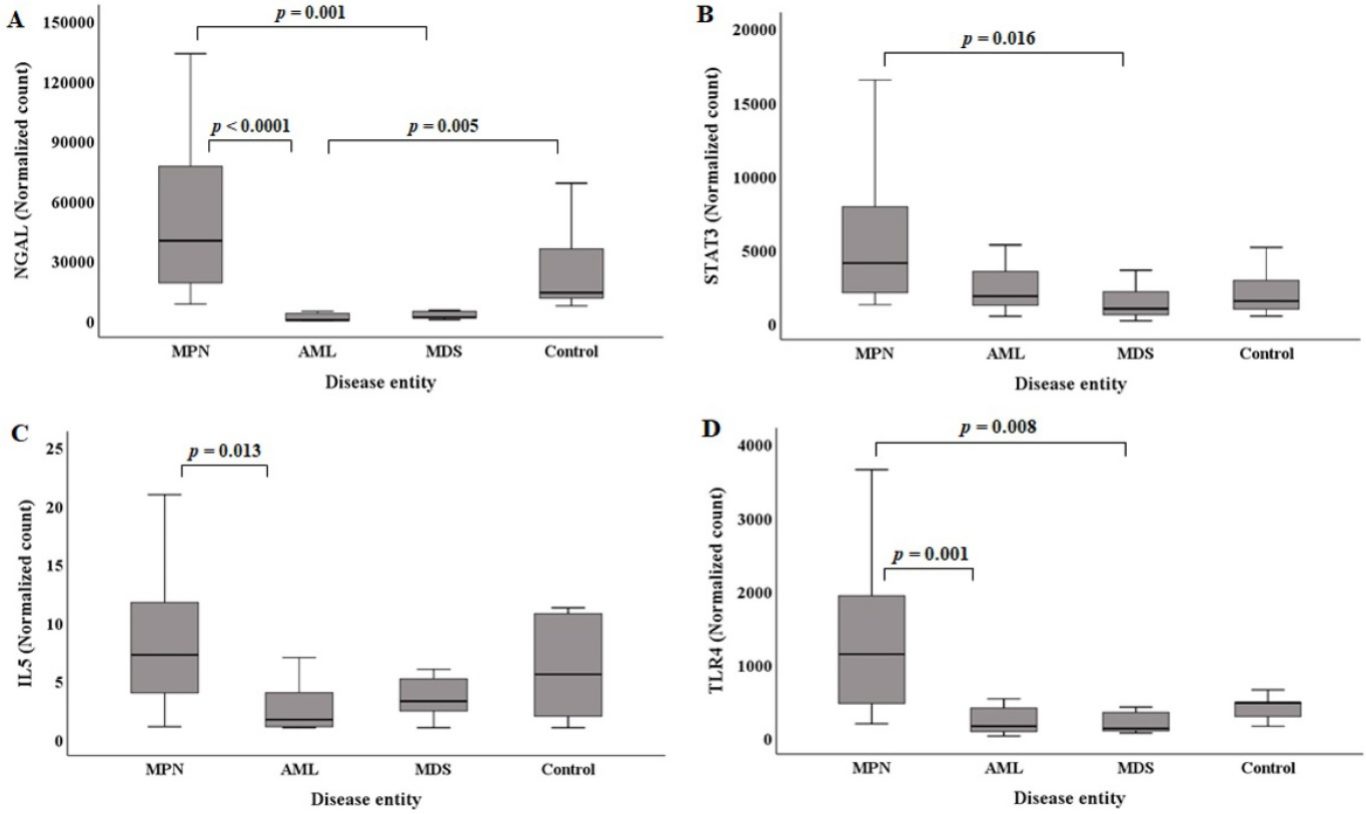
Comparison of *NGAL* (A), *STAT3* (B), *IL5* (C), and *TLR4* (D) normalized counts in BM mononuclear cells of the hematological malignancy and control groups. The control group comprised patients with normal BM. (A) *NGAL* normalized counts in the AML and MDS groups are statistically lower than those in the MPN group. *NGAL* normalized counts in the AML group are statistically lower than those in the control group. (B) *STAT3* normalized counts in the MDS group are statistically lower than those in the MPN group. (C) *IL5* normalized counts in the AML group are statistically lower than those in the MPN group. (D) *TLR4* normalized counts in the AML and MDS groups are statistically lower than those in the MPN group. **Abbreviations**: AML, acute myeloid leukemia; BM, bone marrow; MDS, myelodysplastic syndrome; MPN, myeloproliferative neoplasm; NGAL, neutrophil gelatinase-associated lipocalin.

The median (Q1, Q3) *NGAL* normalized counts according to disease entities are presented in Table 1. The *NGAL* normalized count in the control group was 13965.96 (11045.95, 44238.40). MPN (n=20) showed the highest *NGAL* normalized counts [40032.64 (18328.19, 79735.56)]. The MPN group showed statistically higher *NGAL* normalized counts than the AML and MDS groups (Figure 2A). Acute myeloid leukemia had the lowest *NGAL* normalized counts [211.72 (25.07, 3631.79)]. The AML groups exhibited statistically significant lower *NGAL* normalized counts than the control group (Figure 2A).

The median (Q1, Q3) of *STAT3* normalized counts in the control group was 1506.65 (882.89, 3092.34). MPN (n=20) showed the highest *STAT3* normalized counts [4087.45 (1982.02, 8171.37)]. The MPN group exhibited statistically higher *STAT3* normalized counts than the MDS group (Figure 2B). The MDS group had the lowest *STAT3* normalized counts [982.35 (489.12, 2499.36)].

The median (Q1, Q3) of *IL5* normalized counts in the control group was 5.57 (1.52, 11.02). MPN (n=20) had the highest *IL5* normalized counts [7.25 (3.80, 11.96)]. The MPN group showed statistically higher *IL5* normalized counts than the AML group (Figure 2C). The AML group exhibited the lowest *IL5* normalized counts [1.69 (1.04, 4.57)].

The median (Q1, Q3) of the *TLR4* normalized counts in the control group was 477.72 (270.69, 566.27). MPN (n=20) had the highest *TLR4* normalized counts [1138.15 (393.43, 2012.43)]. The MPN group showed statistically higher *TLR4* normalized counts than the AML and MDS groups (Figure 2D). The MDS group exhibited the lowest *TLR4* normalized counts [127.36 (82.84, 417.55)].

## Discussion

We analyzed the association of cytokines' expressions, using BM MNCs at the diagnostic time of myeloid malignancies, which represent clonal diseases of hematopoietic stem cells (HSCs) 21. BM MNCs include not only hematopoietic progenitor cells at different stages of maturation (drived by HSCs) but also mesenchymal stromal cells (MSCs) comprising the HSC niche 22. HSC niches are local tissue microenvironments that maintain and regulate stem cells, being often located near trabecular bone and created partly by MSCs and endothelial cells 23. Recent studies showed that myeloid malignancies are caused by different genetic and epigenetic changes of HSCs and functional changes in HSC niche cells such as MSCs, while the interplay by various kinds of cytokines between HSCs and the niche play an important role 24. Especially the interplay between both of them were reported to change during disease evolution 24. In this context, our study displayed the association of cytokines in MNCs (including HSCs and MSCs) involved in the interplay at the diagnostic time of myeloid malignancy, especially presenting the association of *NGAL* with the relatively well known cytokine genes such as *STAT3*, *TLR4*, *IL5*, *TLR1* and *TLR2*.

Demographic and clinical data showed that AML and MDS groups had significantly lower Hb levels than the control (Table 1; Supplementary Table S1). Hb, neutrophil counts, platelet counts, and *NGAL* normalized counts were significantly lower in the AML and MDS groups than in the MPN group (Table 1; Supplementary Table S1). *NGAL* normalized counts showed a pattern similar to BM NGAL protein levels in previous studies 8, 10.

CRP levels were significantly higher in the AML group than those in the control group (Table 1; Supplementary Table 1). Although CRP levels (protein levels measured in peripheral blood, Table 1) were higher in the AML group, there was no significant difference between the AML and control groups with respect to *CRP* expression in BM MNCs (Supplementary Table 2). This is because plasma CRP is mainly produced in hepatocytes, but *CRP* gene expression in our study was measured in BM mononuclear cells 25. In line with that, in our study, *CRP* gene showed very low normalized counts among all 49 genes (Table 2), and did not show significant differences between the groups (Supplementary Table 2).

Normalized counts of *NGAL* had the highest statistical association with those of *STAT3* (R^2^=0.799, *p<*0.001) and *TLR4* (R^2^=0.833, *p<*0.001). However, normalized counts of *NGAL* were not significantly associated with those of *BCL2L1, CRP, CXCL2, CASPASE-1, IL10, IL11, IL2, IL28A/B, IL29, IL3, IL6, IL7, INFB, IRF7, NF-kB, RAGE, STAT4,* or* TLR5* (Table 2). In a previous study using human macrophages, IL-10 affected the expression of *NGAL* 26, but its normalized counts in our study did not show an association with the normalized counts of *NGAL*. This will be probably because IL-10 does not directly affect *NGAL* expression 26. IL-10 has been reported to affect *NGAL* expression through the Janus kinase-STAT pathway 26.

Multiple regression model 2 (Table 3) for predicting *NGAL* normalized counts included five independent factors, among which, *STAT3* normalized counts had the highest explanatory power (R^2^=0.799, Table 2). In sequence, *IL5* (R^2^=0.342, Table 2), *TLR1* (R^2^=0.303, Table 2), *TLR2* (R^2^=0.193, Table 2), and *IRAK4* (R^2^=0.170, Table 2) normalized counts exhibited explanatory power. In a previous study using human macrophages, *STAT3* was an important transcription factor for *NGAL* expression 26. Additionally, *NGAL* has been suggested to induce the growth and proliferation of breast cancer cells, lung cancer cells, and hepatoblatoma 26. Similarly, our study based on human BM MNCs showed that *NGAL* normalized counts had a statistically high association with *STAT3* normalized counts. Additionally, *NGAL* and *STAT3* normalized counts showed similar patterns based on disease entity, especially in the MPN and MDS groups (Figure 2 A and B). Further studies including more human BM MNC specimens in several hematological malignancy entities could identify the association between *NGAL* and *STAT3* more clearly, helping to elucidate their developmental mechanisms.

In multiple regression model 2, the* TLR4* normalized count was removed as an independent factor because it exhibited multicollinearity with the *STAT3* normalized count. However, the* TLR4* normalized count was an independent factor with higher explanatory power (R^2^=0.833, Table 2) for the *NGAL* normalized count than the *STAT3* normalized count (R^2^=0.799, Table 2) in simple regression analysis. Additionally, the pattern of *NGAL* normalized counts in the disease and control groups was more similar to that of *TLR4* normalized counts than to that of *STAT3* normalized counts (Figure 2 A, B, and D). Although the relationship between *TLR4* and *NGAL* has been reported in previous studies, it was found in organs or tissues other than BM, such as the urinary system and A549 cells 1, 27. In the urinary system, *TLR4* expression is crucial for the secretion of urinary NGAL but does not directly affect *NGAL* expression 27. In A549 cells (adenocarcinomic human alveolar basal epithelial cells), the transfection of both *TLR4* and its cofactor MD2 led to the upregulation of *NGAL* expression 1. Studies using human macrophages and monocytes showed an association between *TLR4* and *STAT3*, but not a direct relationship of *TLR4* to *NGAL* 28, 29. Previous studies based on human monocytes and macrophages suggested that *TLR4* does not affect *NGAL* expression as directly as *STAT3* 26, 28, 29. Accordingly, *TLR4* normalized counts, but not *STAT3* normalized counts, were finally removed as an independent factor in our multiple regression model.

In multiple regression model 2, *IL5* normalized counts had the second highest explanatory power (R^2^=0.342, Table 2), and if the pattern of the *STAT3* normalized counts was combined with the pattern of *IL5* normalized counts, the combined pattern became more similar to that of the *NGAL* normalized counts (Figure 2 A, B, and C).

There was no sign of severe infections in AML group of our study. Nevertheless, AML group showed high CRP and low NGAL expression (Table 1), which seems contradictory. As for high CRP levels at the diagnostic time of AML, AML patients with high WHO performance score were reported to have higher CRP levels than those with low score (patients with performance score 3/4 had 6.81 mg/dL higher CRP compared to patients with performance score 0), while in that previous study patients who had a bacteraemic episode within 30 days of AML diagnosis were excluded 30.

However, WHO performance scores of AML patients in our study could not be identified accurately from medical records. Only given that four, two and one AML patients had over 70 years of age, PML-RARA rearrangement and brain hemorrhage, respectively, it could be speculated that AML group in our study would have had relatively high WHO scores. This possibly could lead to high CRP in AML group of our study.

As for low NGAL, a previous study using BM supernatant showed that AML group had low NGAL and high CRP levels 8. That study suggested that the reason to low NGAL levels in AML group would be due to neutrophils or neutrophilic precursors as synthesis and storage sites of NGAL being suppressed by increased leukemic cells 8.

Nevertheless, as the limitation of our study, since MNCs were isolated from the BM via density, neutrophils would not have been necessarily preserved in the buffy coat and this could have biased the NGAL expression level in our study (more lymphocytes and less myeloid cells). To prevent such bias, future studies measuring the expression of cytokines and analyzing their association at single cell level are needed, which will also help elucidate the association of cytokines in myeloid malignancy more accurately and specifically.

In conclusion, to the best of our knowledge, the present study analyzed for the first time, the association between the normalized counts of *NGAL* and those of the cytokine genes in human BM MNCs. First, simple regression analysis identified 26 cytokine gene normalized counts that were statistically significantly related to *NGAL* normalized counts, although *STAT3* and *TLR4* normalized counts had the highest explanatory power. Stepwise multiple regression analysis was used to develop a multiple regression model as follows: *NGAL* normalized counts=4316.825 + 9.056 × *STAT3* normalized counts + 844.226 × *IL5* normalized counts + 17.540 × *TLR1* normalized counts - 28.206 × *TLR2* normalized counts - 42.524 × *IRAK4* normalized counts. Multiple regression analysis showed that *STAT3* and *TLR4* normalized counts exhibited multicollinearity and a statistically high association. *STAT3*, *IL5*, and *TLR4* normalized counts showed similar patterns to *NGAL* normalized counts in hematological malignancy and control groups. Future studies are warranted to identify and confirm these relationships more accurately and specifically in hematological malignancy diseases, which would help elucidate their developmental mechanisms.

## Supplementary Material

Supplementary tables.

## Figures and Tables

**Table 1:** Patient demographic features and laboratory parameters of each hematological malignancy and control group (n=48)

	MPN^†^ (n=20)	AML (n=12)	MDS (n=7)	Control^‡^ (n=9)	*p*-value^*^
Gender	10 males, 10 females	11 males, 1 females	2 males, 5 females	8 males, 1 females	
Age	55 (39, 63)	59 (43, 72)	70 (59, 81)	63 (47, 66)	0.335
Hb (g/L)	124 (105, 144)	79 (69, 83)	86 (66, 93)	135 (131, 146)	<0.0001
WBC count (10^9^/L)	19.31 (10.45, 107.23)	3.37 (2.36, 9.61)	2.38 (1.61, 3.12)	6.22 (4.32, 7.30)	<0.0001
Neutrophil count (10^9^/L)	14.43 (7.49, 68.80)	0.74 (0.23, 1.65)	0.50 (0.43, 1.09)	3.78 (2.55, 4.31)	<0.0001
Platelet count (10^9^/L)	620 (404, 849)	49 (24, 116)	78 (52, 127)	255 (209, 300)	<0.0001
CRP (mg/dL)	0.100 (0.030, 0.465)	8.390 (1.202, 12.255)	0.400 (0.324, 0.845)	0.080 (0.058, 0.161)	0.001
eGFR^||^ (mL/min/1.73 m^2^)	94.50 (79.81, 109.68)	86.72(69.36, 94.50)	80.95 (66.37, 99.18)	92.15(85.24, 102.29)	0.453
*NGAL* gene normalized counts	40032.64 (18328.19, 79735.56)	211.72 (25.07, 3631.79)	1638.79 (1129.19, 4946.41)	13965.96 (11045.95, 44238.40)	<0.0001

Quantitative data are presented as the median [quartile1 (Q1), Q3] values; ^†^MPN included CML (n=8), PV (n=4), ET (n=4), PMF (n=3), and MPN-U (n=1); ^‡^The control group comprised patients with lymphoma without BM involvement (n=7) or normocellular marrow without hematological malignancy according to the BM smear and pathological review (n=2);^ *^for age and Hb variable, the one-way ANOVA test was performed, and for WBC count, neutrophil count, platelet count, CRP, eGFR, and *NGAL* gene normalized count variable, the Kruskal-Wallis H-Test was performed; ^||^, eGFR was calculated using the CKD-EPI equation. **Abbreviations**: AML, acute myeloid leukemia; BM, bone marrow; CKD-EPI, Chronic Kidney Disease Epidemiology Collaboration equation; CML, chronic myeloid leukemia; CRP, C-reactive protein; eGFR, estimated glomerular filtration rate; ET, essential thrombocythemia; Hb, hemoglobin; MDS, myelodysplastic syndrome; MPN, myeloproliferative neoplasm; MPN-U, MPN-unclassifiable; PMF, primary myelofibrosis; PV, polycythemia vera; WBC, white blood cell.

**Table 2:** Simple regression analysis of normalized counts of *neutrophil gelatinase‐associated lipocalin* (*NGAL*) and 48 cytokine genes in 48 bone marrow mononuclear cells

Gene Name	R^2^	*P*-value^†^	Normalized counts^‡^
*BAX*	0.361	<0.001	430.30 (129.17, 921.12)
*BCL2L1*	0.005	**0.650**	1161.90 (445.83, 3083.26)
CASP8	0.602	<0.001	1829.57 (1060.55, 3199.58)
*CRP*	0.004	**0.657**	1.15 (1.00, 2.41)
*CXCL1*	0.405	<0.001	41.38 (20.53, 149.78)
*CXCL10*	0.085	0.044	4.26 (1.19, 16.08)
*CXCL2*	0.002	**0.792**	65.42 (28.43, 235.26)
*Caspase-1*	0.010	**0.508**	140.78 (51.02, 600.28)
*ELK1*	0.407	<0.001	44.86 (31.60, 86.38)
*FOS*	0.252	<0.001	1907.91 (822.54, 5739.86)
*IL10*	0.004	**0.669**	4.30 (1.96, 9.44)
*IL11*	0.033	**0.218**	4.15 (1.91, 5.64)
*IL18*	0.495	<0.001	160.25 (71.47, 346.07)
*IL1A*	0.506	<0.001	4.60 (1.75, 8.30)
*IL1B*	0.095	0.033	36.69 (16.44, 157.54)
*IL1R1*	0.550	<0.001	19.69 (7.74, 71.20)
*IL1R2*	0.537	<0.001	313.50 (45.26, 1513.25)
*IL2*	0.017	**0.373**	2.62 (1.05, 4.12)
*IL28A/B*	0.001	**0.864**	1.62 (1.01, 2.87)
*IL29*	0.001	**0.803**	1.00 (1.00, 1.16)
*IL3*	0.001	**0.840**	1.10 (1.00, 1.39)
*IL4*	0.187	0.002	14.08 (6.35, 45.30)
*IL5*	0.342	<0.001^*^	4.63 (1.86, 10.76)
*IL6*	0.002	**0.791**	1.17 (1.00, 2.75)
*IL7*	0.021	**0.328**	10.47 (4.38, 19.29)
*IL8*	0.336	<0.001	488.11 (140.69, 2125.02)
*IL9*	0.462	<0.001	1.15 (1.00, 2.03)
*INFB*	0.003	**0.704**	3.28 (1.85, 4.99)
*IRAK1*	0.549	<0.001	444.39 (268.83, 743.36)
*IRAK2*	0.223	0.001	57.38 (36.08, 159.63)
*IRAK4*	0.170	0.004^*^	182.62 (51.42, 431.18)
*IRF7*	0.065	**0.080**	110.71 (43.56, 228.80)
*MyD88*	0.262	<0.001	616.36 (276.34, 1634.68)
*NF-kB*	0.027	**0.264**	41.94 (18.64, 131.04)
*NFKB1A*	0.127	0.013	1744.90 (772.39, 4030.14)
*NLRP3*	0.215	0.001	344.61 (120.15, 766.43)
*RAGE*	0.001	**0.798**	22.01 (8.80, 54.45)
*STAT1*	0.103	0.026	767.49 (378.39, 1864.03)
*STAT2*	0.236	<0.001	858.11 (479.27, 1579.37)
*STAT3*	0.799	<0.001^*^	2387.19 (1319.69, 4416.35)
*STAT4*	0.023	**0.309**	213.48 (53.50, 467.81)
*TLR1*	0.303	<0.001^*^	266.94 (117.14, 819.62)
*TLR2*	0.193	0.002^*^	356.11 (171.76, 737.90)
*TLR4*	0.833	<0.001^*^	425.17 (184.17, 1145.19)
*TLR5*	0.048	**0.136**	18.35 (6.64, 47.35)
*TNF*	0.097	0.031	115.23 (51.74, 295.85)
*TRAF3*	0.721	<0.001	501.40 (304.37, 891.38)
*TRAF6*	0.271	<0.001	119.15 (59.24, 288.61)

^†^ Adjusted alpha was determined as 0.026;^ ‡^Normalized counts are presented as the median [quartile1 (Q1), Q3] values; ^*^ they were indicated, because those cytokine genes were included as independent factors in multiple regression models; *P*-values > 0.05 were typed in boldface. **Abbreviations**: AML, acute myeloid leukemia; BAX, bcl-2-associated X protein; BCL2L1, bcl-2-like 1; CXCL, chemokine (C-X-C motif) ligand; *ELK1*, *ETS like transcription factor-1*; IL, interleukin; IL1A, IL 1 alpha; IL1R2, IL1 receptor, type 2; *IRF7*, *interferon regulatory factor 7*; MDS, myelodysplastic syndrome; *NGAL*, *neutrophil gelatinase-associated lipocalin*; Q, quartile; RAGE, receptor for advanced glycation end products; TLR4, Toll-like receptor 4.

**Table 3:** Regression analysis models of the relationship between the normalized counts of *NGAL* and those of cytokine genes in the bone marrow

	Coefficient	*t*‐value	*P*‐value^†^	VIF	Adj *R*^2^	AIC
Model 1						
Constant	4165.283	1.718	0.093		0.936	885.642
*TLR4*	7.676	1.602	0.117	17.277		
*IL5*	882.859	3.769	0.001	1.374		
*STAT3*	6.792	4.532	<0.001	22.683		
*TLR2*	-27.477	-5.789	<0.001	5.534		
*TLR1*	16.348	4.567	<0.001	8.671		
*IRAK4*	-35.902	-4.424	<0.001	4.539		
						
Model 2						
Constant	4316.825	1.749	0.088		0.933	886.557
*IL5*	844.226	3.558	0.001	1.359		
*STAT3*	9.056	17.792	<0.001	2.522		
*TLR2*	-28.206	-5.862	<0.001	5.484		
*TLR1*	17.540	4.919	<0.001	8.296		
*IRAK4*	-42.524	-5.979	<0.001	3.362		

**Abbreviations**: Adj, adjusted; AIC, Akaike's information criterion; VIF, variance influence factor. ^†^Statistically significant (*P<*0.05).

## Abbreviations

Adj: adjusted
AIC: Akaike's information criterion
AML: acute myeloid leukemia
BM: bone marrow
BAX: bcl-2-associated X protein
BCL2L1: bcl-2-like 1
CKD-EPI: Chronic Kidney Disease Epidemiology Collaboration equation
CML: chronic myeloid leukemia
CRP: C-reactive protein
CXCL: chemokine (C-X-C motif) ligand
DEG: differentially expressed gene
eGFR: estimated glomerular filtration rate
ELK1: ETS like transcription factor-1
ET: essential thrombocythemia
Hb: hemoglobin
IL: interleukin
IL1A: IL 1 alpha
IL1R2: IL1 receptor, type 2
IRF7: interferon regulatory factor 7
MDS: myelodysplastic syndrome
MNC: mononuclear cell
MPN: myeloproliferative neoplasm
MPN-U: MPN-unclassifiable
NGAL: neutrophil gelatinase-associated lipocalin
PMF: primary myelofibrosis
PV: polycythemia vera
Q: quartile
RAGE: receptor for advanced glycation end-products
TLR4: Toll-like receptor 4
VIF: variance influence factor
WBC: white blood cell
