# Supplementary material for: Analysis of the Relationship between the Expression Levels of Neutrophil Gelatinase-Associated Lipocalin and Cytokine Genes in Bone Marrow
Source: Int J Med Sci. 2021 Jul 23;18(14):3290–8. doi: 10.7150/ijms.62425 (PMC8364451; doi:10.7150/ijms.62425)
Supplement: Supplementary file 1 — Supplementary tables. [file ijmsv18p3290s1.pdf]

Supplementary Table S1. Pair-wise comparison of six numerical variables in four (MPN, AML, MDS, and Control) groups.

Variable	Comparison groups	*p*-value*
Hb	MPN vs. AML	<0.0001
	MPN vs. MDS	0.002
	AML vs. Control	<0.0001
	MDS vs. Control	0.001
WBC count	MPN vs. MDS	<0.0001
Neutrophil count	MPN vs. AML	<0.0001
	MPN vs. MDS	<0.0001
Platelet count	MPN vs. AML	<0.0001
	MPN vs. MDS	0.001
CRP	MPN vs. AML	0.002
	AML vs. Control	0.003
NGAL normalized counts	MPN vs. AML	<0.0001
	MPN vs. MDS	0.001
	AML vs. Control	0.005

* Adjusted alpha was determined to be 0.006 after Bonferroni correction.

Abbreviations: AML, acute myeloid leukemia; CRP, C-reactive protein; Hb, hemoglobin; MDS, myelodysplastic syndrome; MPN, myeloproliferative neoplasm; NGAL, neutrophil gelatinase-associated lipocalin; WBC, white blood cell.

Supplementary Table S2. Normalized counts of *CRP* gene in bone marrow mononuclear cells of each hematological malignancy and control group

Gene name	MPN† (n=20)	AML (n=12)	MDS (n=7)	Control‡ (n=9)	*p*-value*
CRP	1.60 (1.05, 2.57)	1.00 (1.00, 1.34)	1.00 (1.00, 3.98)	2.20 (1.02, 3.24)	0.091

Quantitative data are presented as the median [quartile1 (Q1), Q3] values; †MPN included CML (n=8), PV (n=4), ET (n=4), PMF (n=3), and MPN-U (n=1); ‡The control group comprised patients with lymphoma without BM involvement (n=7) or normocellular marrow without hematological malignancy according to the BM smear and pathological review (n=2); *The Kruskal-Wallis H-Test was performed.

Abbreviations: AML, acute myeloid leukemia; BM, bone marrow; CML, chronic myeloid leukemia; CRP, C-reactive protein; ET, essential thrombocythemia; MDS, myelodysplastic syndrome; MPN, myeloproliferative neoplasm; MPN-U, MPN-unclassifiable; PMF, primary myelofibrosis; PV, polycythemia vera.
